# Relationship between Live-In Grandparents and Grandchild’s Health and Well-Being in Palestinian Refugees in Lebanon

**DOI:** 10.3390/ijerph20010370

**Published:** 2022-12-26

**Authors:** Olfat B. Sheikomar, Hala Ghattas, Nadine R. Sahyoun

**Affiliations:** 1 Department of Nutrition and Food Science, College Park, University of Maryland, College Park, MD 20742, USA; 2 Department of Food and Nutrition, King Abdulaziz university, Jeddah 21589, Saudi Arabia; 3 Center for Research on Population and Health, American University of Beirut, Beirut 1107 2020, Lebanon; 4 Arnold School of Public Health, University of South Carolina, Columbia, SC 29208, USA

**Keywords:** grandparents, grandchildren, mental wellbeing, health, acute illness, chronic disease, school attendance, child employment, food insecurity

## Abstract

Grandparents (GP) play influential roles in grandchildren’s health, behavior, and life. However, this relationship has not been examined in the Arab region. This study assesses whether the presence of GP in the household is associated with grandchildren’s health and wellbeing. Health status was determined by a child experiencing chronic health conditions or an acute illness, and wellbeing was determined based on school attendance and child labor. Data were collected through surveys conducted in 2010 and 2015 of representative samples of Palestinian refugees living in Lebanon. Multivariate logistic regression showed that, even after controlling for potential confounders, including the presence of parents in the household and household food insecurity (FI), the presence of live-in GP was associated with lower odds of children experiencing acute illnesses (OR 0.74 95% CI 0.62–0.92) and higher odds of attending school (OR 2.22 95% CI 1.28–5.33), but not child labor. The presence of GP in the household may be protective to grandchildren’s health status and school attendance in this population.

## 1. Introduction

Palestinian refugees constitute one of the world’s largest displaced populations [1]. Even though they have been displaced since 1948 and have a long-standing presence in Lebanon, they are a marginalized population because of their socio-economic deprivation, restrictions on their human rights, and exclusion from mainstream life [2]. These conditions make them vulnerable to inadequate access to health care, education, and food, and consequently increased experience of food insecurity (FI) [3]. This is especially true, as Palestinian refugees in Lebanon require special work permits that are hard to obtain, and they are restricted to certain non-professional or not well paid employment [4]. They are also not allowed to run a business or own property and cannot access public services. Most Palestinian refugees living in camps depend to a great extent on humanitarian assistance from the United Nations Relief and Works Agency for Palestine Refugees in the Near East (UNRWA) [1,4]. UNRWA is unique in terms of its long-standing commitment to one group of refugees, whereby it provides human development and humanitarian services, encompassing education, primary health care, welfare relief and social services, infrastructure and camp improvement, microfinance, and emergency assistance [5].

Palestinian refugee children are especially at risk in the camps, as they live in overcrowded quarters, which lack resources, and many are exposed to ongoing conflict, violence, and discrimination [4,6]. Despite these challenging conditions, many families have lived there for more than 70 years in multigenerational families [7]. In refugee camps, several factors converge to contribute to the family structure, which involves grandparent (GP) co-residence, such as gathering in close quarters and in specific camps, economic disparities and disruptions, health problems, and financial strain [2,4]. The formation of multigenerational families may be related to loss of a parent of children or maternal employment. In such households, GP are often involved in raising children and in participating in the health and wellbeing of their grandchildren [7,8,9]. A child’s wellbeing means the quality of a child’s life, measured using health status, educational attainment, and socio-economic status [10]. Involvement of GP could positively impact grandchildren, as they can serve as role models, discuss appropriate behavior, encourage academic and health-related activities, provide advice, financial or emotional support, and prepare food. Alternatively, GP could negatively impact their grandchildren by spoiling and indulging them [11], or GP could be in poor health, which would place a financial burden on the family. There is no data in the Arab world on the number of grandchildren being raised by their GP. In the United States, more than 13 million children live in homes with their GP, and 2.5 million GP take primary responsibility for raising these children [12].

Previous studies mainly focused on the impact that raising grandchildren may have on the health and wellbeing of GP [13,14,15,16,17,18], but a few studies have also assessed the influence of GP on grandchildren’s health and wellbeing. A study conducted in England showed that a high level of grandparental involvement increased the wellbeing of children and decreased their emotional and behavioral problems [14]. A review by Dunifon showed mixed results of the influence of live-in GP on children’s wellbeing in the U.S. Some of the studies showed positive associations represented by prosocial behaviors, academic engagement, self-confidence, positive mental health, and maturity, while other studies showed more significant behavioral problems, lower educational attainment, and physical and mental health issues among the grandchildren [11]. It was suggested that these relationships may be influenced by GP’s educational attainment [19].

Other factors may also influence the relationship between GP and their grandchildren, such as poverty and household FI, and these in turn affect children’s health and wellbeing. Ziliak et al. used longitudinal data from the 2001 to 2010 Current Population Survey in the U.S. and found that the prevalence of FI in families with a grandchild is at least two times higher compared to families without a grandchild [20]. It is also possible that children’s health and wellbeing may be influenced by the GP’s age, socio-economic status, family structure, child’s gender, and the number of children in the household [21,22]. GP’s good health status may also provide an impetus for taking care of grandchildren [17].

To our knowledge, the association between a live-in GP and grandchildren’s health and wellbeing has not been assessed, neither in the Arab population, nor among refugees. Therefore, using data from two surveys of representative samples of Palestinian refugees in Lebanon, this study examines this association between GP and the health and wellbeing of children in the household.

## 2. Materials and Methods

### 2.1. Study Design

Data for this study were obtained from two surveys conducted by the American University of Beirut and funded by UNRWA. The surveys were conducted in July-August 2010 and in April 2015 targeting Palestinian refugees living in the 12 official Palestinian refugee camps and gatherings outside the camps in Lebanon [4,6]. The sampling frame for the 2010 survey was based on the Palestinian refugee’s list from the UNRWA Refugee Registration and Information System, while the sampling frame for the 2015 survey used the list of Palestinian refugees in Lebanon compiled by the Palestinian Central Bureau of Statistics in 2010. In both surveys, a representative sample of households was obtained using a stratified multistage cluster sampling. A simple random approach was used for the data collected from camps and a snowball sampling technique for the data collected from non-camp gatherings [4,6].

#### 2.1.1. Sociodemographic and Economic Variables

Data were collected using a questionnaire developed by the research team and translated into Levantine Arabic dialect. The questionnaire was administered through face-to-face interviews with a proxy adult responsible for food preparation, preferably a woman in the selected household. If a woman was not present, any adult family member was interviewed and served as a proxy to collect information on all household members. The questionnaires used in both surveys were similar. The first section of the questionnaire collected information at the household level, including household size, number of adults, and number of children, household expenditure on health care and on food, and the receipt of welfare programs and health care services. A food-related asset scale was calculated based on the ownership of a refrigerator, freezer, oven, and microwave, each contributing one point to the scale. Food security status was measured using the modules shown in Table 1. The food security modules were slightly different by survey year; however, these were calibrated, and the scores were adjusted. The second part of the questionnaire was designed to obtain information on each household member, including age, marital status, educational attainment, and relationship to the head of household.

#### 2.1.2. Outcome Variables

The outcome variables measured included: school attendance, child labor, and child health status. School attendance was determined using the question “Did (name) enroll for the current school year?”. Response options included “yes” if the response was “Enrolled and attend the school” and “No” if the responses were “Enrolled but not attending, enrolled but dropped out, or not enrolled.”. Child labor was defined using the question “Did (name) work for wage (cash or in kind) even for one hour during last week?”. Response options were “employed” if the response was yes and “unemployed” if the response was no or work without pay. Health status was based on having a chronic disease or an acute illness within the past 6 months. The questions were “has (name) been diagnosed with a chronic disease?” and “has (name) been diagnosed with an acute illness in the past 6 months?”. Response options for both questions were yes or no. Written or oral consent was obtained from individuals before participation in the survey. The studies were approved by the American University of Beirut Institutional Review Board.

For this study, the final analytical sample was composed of 2707 households with at least one child and with no missing information on household food security status. Additionally, the total number of children included in this study was 8034 children between the ages of 0 and 17 years.

### 2.2. Statistical Analysis

Descriptive characteristics were stratified by households with and without live-in GP. A T-test for continuous variables and chi-square for categorical ones were conducted to evaluate differences in characteristics of households and of children with and without a live-in GP. Multivariate logistic regressions were conducted to examine the relationship between children living with GP and the health and wellbeing-related variables of children. Health status was determined based on a child experiencing an acute illness or chronic disease, and wellbeing was measured as school attendance during the previous year and employment status based on the previous week. All regression models were adjusted for potential confounding variables, including child’s sex, age group, child health insurance, and household food security, presence of a parent, grandparent with one or more chronic health condition, household receiving welfare, health expenditure per capita, and household size.

Data were analyzed using SAS (version 9.4; SAS Institute Inc., Cary, NC, USA). All analyses were weighted to adjust for complex survey design. Household ID and survey year were added to the models as fixed effects. All associations were considered statistically significant at *p* < 0.05.

## 3. Results

### 3.1. Characteristics of Households and of Children with and without a Live-In Grandparent

This study included data from 2707 households and 8034 children between the ages of 0 to 17 years. Of these households, 214 had live-in GP and most children lived in households with no GP (Table 2 and Table 3). Compared to households without GP, those with GP had significantly higher prevalences of severe FI, poverty, receiving welfare, including younger mothers and younger fathers, and having fewer working mothers (Table 2). The mean household size, number of adults in the household, healthcare expenditure per capita, and food-related assets were all significantly higher for households with a live-in GP. However, the mean number of children and household food expenditure per capita were significantly lower for households with live-in GP. Additionally, children living in a household with a live-in GP were younger, lived in poorer households, and in households with higher severe FI. Although only 30.8% of households with a live-in GP had a parent living in the household, the majority of children who lived with a GP also had a parent living in the household (69.5%) (Table 3).

### 3.2. Multivariate Factors Associated with Child Health and Wellbeing

The odds of a child with an acute illness were significantly lower in households with a live-in GP (OR 0.74, 95% CI 0.62–0.92) and in households with a parent (OR 0.73, 95% CI 0.40–0.93). Additionally, the odds were lower among female children than males (OR 0.87, 95% CI 0.82–0.93) and with increasing household size (OR 0.89, 95% CI 0.84–0.95) (Table 4). In contrast, the odds of having an acute illness were significantly higher for children aged 0–4 years compared to children 5–11 years (OR 1.35, 95% CI 1.16–1.57) in those living in severely food insecure households (OR 1.45, 95% CI 1.17–1.80), receiving welfare (OR 1.39, 95% CI 1.17–1.65), and was also higher in households with higher healthcare expenditure per capita (OR 1.42, 95% CI 1.18–1.26) (Table 4).

The odds of having a chronic disease were also lower for female children (OR 0.75, 95% CI 0.65–0.87), children aged 0–4 years compared to children aged 5–11 years (OR 0.61, 95% CI 0.49–0.76), and decreased with increasing household size (OR 0.91, 95% CI 0.84–0.98). Similarly, the odds of having a chronic disease were higher for children in severely food insecure households (OR 1.56, 95% CI 1.19–2.07), households receiving welfare (OR 1.54, 95% CI 1.22–1.94), and increased with increasing healthcare expenditure per capita (OR 1.45, 95% CI 1.14–1.86).

Children were more likely to attend school if they were from a household with a live-in grandparent (OR 2.22, 95% CI 1.28–5.33), lived with at least a parent in the household (OR 1.75, 95% CI 1.59–2.20), were female (OR 1.44, 95% CI 1.04–1.98), aged 6–12 years (OR 3.67, 95% CI 2.41–4.64), and from households receiving welfare (OR 1.37, 95% CI 1.07–1.94).

Children in severely food insecure households (OR 0.68, 95% CI 0.35–0.71) were less likely to attend school. Additionally, the odds of school attendance were lower with higher healthcare expenditure per capita (OR 0.81, 95% CI 0.66–0.99). Children in food insecure households were more likely to be employed in child labor (OR 1.83, 95% CI 1.30–2.78). The likelihood of child labor also increased with household size (OR 1.95, 95% CI 1.86–2.04). In contrast, the odds of child labor were lower for children in households with a parent (OR 0.51, 95% CI 0.46–0.69), who were female (OR 0.72, 95% CI 0.59–0.89), aged 5–14 years (OR 0.27, 95% CI 0.18–0.41), and for those in households receiving welfare (OR 0.62, 95% CI 0.46–0.83) (Table 5).

## 4. Discussion

This study assessed the relationship between the presence of a live-in GP in the household and grandchildren’s health and wellbeing, controlling for potential confounders, including household food security and the presence of parents. Results show that, although households with a live-in GP were of lower socioeconomic status, with higher levels of FI and poverty than households without a live-in GP, children in these households had significantly lower acute illnesses and higher levels of school attendance. The association between the presence of a GP in grandchildren’s lives and the children’s health status showed mixed results in previous studies. Although no studies examined acute illness as an outcome, a few studies examined the relationship between the presence of GP and various health outcomes in children. For example, a longitudinal study found that the involvement of maternal grandmothers was associated with lower mortality rates for children in rural Gambia [24]. In a cross-sectional study of 199 Hispanic American elementary-aged children, Pulgaron and colleagues found that having a GP involved in caretaking was associated with healthy BMI scores for grandchildren [25]. Moreover, a systematic review aimed at assessing the influence of GP on some health risk factors associated with cancer, such as weight, diet, physical activity, and tobacco use found that overall GPs had a positive and beneficial impact on their grandchildren’s cancer risk factors [26]. For psychological health outcomes, Ruiz and Silverstein used data from the second wave of the National Survey of Families and Households (NSFH) and also found that, among youth aged 18–23, grandchild-reported closeness with GP was associated with lower levels of depression [27]. Additionally, among Latino families living in the US, Xie et al. found that GP encouraged their grandchildren to participate in various physical activities, which, in turn, improved grandchildren’s physical and mental health [28].

However, other studies have found adverse health outcomes in children with GP involvement. For example, studies found an adverse effect between GP involvement and higher weights in children [29,30,31,32,33]. Additionally, in a cross-sectional study conducted in a Japanese rural town, Urita et al. found elevated rates of helicobacter pylori transmission from live-in grandmothers to children [34]. Relatively few studies have examined the effects of GP involvement on children’s health outcomes and, therefore, the degree of their influence remains unclear and differ by study design, outcomes measured, and cultural norms [35]. This was also highlighted in a systematic review by Sadruddin et al. [36].

Our study also showed that grandchildren living in a household with GP tended to have higher levels of school attendance. These results support the findings by Zeng et al., who showed that, in rural China, a live-in GP directly affected the educational attainment of their grandchildren compared to non-live-in or deceased GP [37]. Another study found that, in a sample of Muslim and Hindu families, children exhibited better behavioral adjustment when GP were involved in their care [38]. Other studies have shown no relationships or negative ones with a GP living in the household. For example, a longitudinal study using nationally representative survey data on three generations in the Netherlands found no associations between grandchildren’s educational attainment and grandparental involvement in their lives, even after controlling for the strength of grandparental involvement [39]. Another cross-sectional study using data from a nationally-representative group of youth aged 14–19 in the US [40] found that GP had no influence on self-reported grades, risky behaviors such as smoking cigarettes, drinking alcohol or using marijuana, and on sexual behavior [40]. Finally, in a cross-sectional study of a large nationally representative sample of children aged 3 to 17, the results found that children raised by GP were more likely to have had adverse experiences, such as child temperament, attention-deficit/hyperactivity disorder, and caregiver aggravation [41]. It is possible that, in some of these studies, the outcomes examined were not those that can be influenced by GP involvement.

Our study showed that the presence of at least one parent in the household was independently and significantly associated with lower acute illnesses, higher school attendance, and lower child employment. This result was expected as the main role of parents is to meet all the economic, psychological, and health needs of their children regardless of any external support [39]. This result is also in line with a previous longitudinal study which found that the parent’s presence, controlling for their sociodemographic characteristics, were associated with their children’s schooling, but not necessarily the presence of GP [39]. In our study, children living with a single mother was associated with lower odds of school attendance (data not shown). This result is also supported by other studies that showed that living in a single-parent household was associated with lower educational achievement [42,43].

Our study also found that children living in food insecure and poor households were at higher risk of acute and chronic health conditions and were less likely to attend school and more likely to be employed. The overall high poverty and unemployment rates in Palestinian refugee camps contribute to the difficulties that food-insecure households face. Healthcare expenditures place an additional burden on the household budget. GP have a high prevalence of chronic conditions, which may further contribute to FI. These results are supported by previous findings that showed a negative association between FI and various adverse health outcomes [44,45,46,47,48]. Other studies found that FI was associated with low academic performance in children. For example, a systematic review of 23 peer-reviewed articles across western industrialized countries showed negative associations between FI and children’s behavioral, academic, and emotional problems, even after controlling for sociodemographic characteristics [49]. Child labor is higher among refugee children in Lebanon, as compared to Lebanese children, and has previously been shown to be associated with FI [50].

There were no associations between GP having chronic conditions and children’s health and wellbeing. Since GP with chronic conditions are less able to take care of their grandchildren, it was expected that having a live-in GP with chronic conditions would be negatively associated with children’s health and wellbeing. This was shown in a review conducted by Grinstade et al.—that older GP and those with poor health were less able to take care of their grandchildren [18]. In addition, another two cross-sectional studies found that most of the caregivers who reported multiple diagnoses for chronic conditions, such as hypertension, diabetes, heart disease, and mobility issues had difficulties taking care of their grandchildren [51,52]. The lack of the association in our study could be due to the high prevalence of GP with chronic conditions (88.8%), which might mask the association but explain the higher health expenditure in households with a live-in GP. 

Our study showed that boys were more likely to be employed in child labor, while significantly more girls attended school. Similar results were reported in the 2015 Child Labor Survey in Lebanon [50]. The percentage of child labor is higher among boys than girls, while a higher percentage of girls are involved in household chores than boys [50]. Child labor in Lebanon is a serious problem, considering that poverty is highly prevalent in Palestinian camps in Lebanon. We also found that, as the household size increased, the odds of acute illness and chronic disease were lower, but the odds of child labor were higher, as a reflection of the higher financial needs of the household.

In our study, younger children living in refugee camps were more vulnerable to acute illnesses than older children. This could be due to the weaker immune system of the children of this age generally and being exposed to potentially unclean water sources in the camps. These conditions may exacerbate their vulnerabilities to several acute health conditions, such as diarrhea and other infections [4,53]. Additionally, as expected, younger children were more likely to attend school and less likely to be employed in child labor as compared to older children. This may be true as older children may be relied on to contribute to household income if needed, and UNRWA provides free schooling for Palestinian refugee children from grade 1 through 9. UNRWA offers limited free secondary education to Palestinian refugees in Lebanon, making going to school unaffordable for older children [50]. While job opportunities increase as children become older, children tend to work instead of going to school [50]. In our sample, more children in households that received welfare attended school, and fewer were employed in child labor. It is possible that having some financial support allowed for more funding for education for the children.

## 5. Conclusions

This is the first study to our knowledge that assessed the relationship between live-in GP and the health and wellbeing of their grandchildren in the Arab world. The dataset used for this study contains information on all household members, which allowed us to examine relationships between members of the households. However, our study also has some limitations. These results may not be generalizable to other Arab countries, as the Palestinian refugees are a vulnerable population whose living arrangements may not necessarily reflect those households in the Arab countries. Additionally, the intent of this survey was not to analyze the relationship between GP and their grandchildren and, therefore, the data did not contain information about the role of the GP in the household, which is as a caretaker or a caregiver. There also was not enough information about the quality of the GP–grandchild relationship. Such information may play an important role in the strength and the extent of the association between the presence of GP and the health and wellbeing of the grandchild [40]. This may also be true for the gender of the GP, which may impact the type of relationship established with children, however, our numbers were too small to conduct separate analysis by gender. Additionally, although relationships were observed in this study, it is possible that the outcomes examined here, such as experiencing acute illnesses or chronic diseases, school attendance, and child labor, may not be those that are most influenced by GP involvement. It has actually been suggested that the heterogeneity found in terms of how GP influence their grandchildren’s health and development may depend on how much contact GP have with their grandchildren, the type and extent of their involvement in caregiving, and GP contribution in terms of financial support [36]. It remains to be seen whether a fuller set of grandchildren outcomes may be more influenced by grandparental involvement, type, and extent of involvement, and whether involvement plays a different role at different life course stages.

Because the role of GP in the lives of grandchildren is such an understudied topic, more work is needed to reinforce these findings and further illuminate the relationships examined here. This is of importance as older adults are living longer and take a more active role in the lives of their grandchildren and support the family as both parents are working. GP have and continue to play an important role in grandchild rearing, as was witnessed in the last decades when mortality from AIDS/HIV was at its peak. Clearly, the types of relationships that exist and the impact of these relationships on grandchildren are culturally driven and must be examined in such context. Additionally, the impact of these relationships may also affect the health and wellbeing of the GP as well.

## Figures and Tables

**Table 1:** Measurement tools used to assess food security (FS) among Palestinian refugees in the 2010 and 2015 Socio-Economic Surveys of Palestinian Refugees in Lebanon *.

Item Label	Palestinian Refugee Survey 2010 ^a^	Arab Family Food Security Scale (AFFSS) 2015 ^b^
1. Inadequate quality food (food sufficiency question) ^1^	Which of these sentences applies the most to the food eaten by your household during the past 6 months? We had enough to eat of the kinds of food we wanted We had enough to eat but not always the kinds of food we wanted Sometimes we did not have enough to eat Often, we did not have enough to eat	Which of these sentences applies the most to the food eaten by your household during the past 6 months? We had enough to eat of the kinds of food we wanted We had enough to eat but not always the kinds of food we wanted Sometimes we did not have enough to eat Often, we did not have enough to eat
2. Concerned food would run out ^2^		In the last 6 months, was there a time when you were concerned that you would run out of food for your household for the next month? (Yes/No)
3. Food bought didn’t last ^2^	Tell me if this statement applies to you most of the time, sometimes, or never: “The food that we bought did not last us and we didn’t have money to buy more”	In the past 6 months, has it ever happened that the food you bought was not enough and you didn’t have money to buy more? (Yes/No)
4. Not enough of some foods ^2^	Are there any foods you feel your family does not eat enough of? (Yes/No) If yes, specify:	Were there any foods you feel your family did not eat enough of in the last 6 months? (Yes/No) If yes, specify:
5. Cut size of meal ^3^	In the past 6 months, did you or any other adult in your household ever cut the size of your meal because there was not enough food? (Yes, almost every month, yes, but not every month, yes in only 1 or 2 months, never)	In the past 6 months, did you or any other adult in your household ever cut the size of your meal because there was not enough food? (Yes/No)
6. Skipped meal ^3^	In the past 6 months, did you or any other household members ever skip a meal because there was not enough food? (Yes, almost every month, yes, but not every month, yes in only 1 or 2 months, never)	In the past 6 months, did you or any other household members ever skip a meal because there was not enough food? (Yes/No)
7. Did not eat whole day or went to bed hungry ^3^	In the past 6 months did you or any member in your household not eat for a whole day or go to bed hungry because there was not enough food? (Yes, almost every month, yes, but not every month, yes in only 1 or 2 months, never)	In the past 6 months did you or any member in your household not eat for a whole day or go to bed hungry because there was not enough food? (Yes/No)

* Source: Adapted with permission from Sahyoun et al. 2014, [23]. ^a^ Household FS status was classified based on the total score as food secure (0–1), moderately food insecure (2–4), and severely food insecure (5–6). ^b^ Household FS status was classified based on the final score as food secure (0–2), moderately food insecure (3–5), and severely food insecure (6–7). ^1^ Item 1 was coded as affirmative for all responses except for first response option. ^2^ Items 2, 3, and 4 were coded as affirmative for yes responses. ^3^ Items 5, 6, and 7 were coded as affirmative for any of the three yes responses.

**Table 2:** Sociodemographic and economic characteristics of households with children with and without a live-in grandparent (GP), 2010 and 2015 Socio-Economic Surveys of Palestinian Refugees in Lebanon surveys. * ^1^.

Characteristics at the Household Level	Household with a Live-In GP n = 214	Household without a Live-In GP n = 2493	*p*-Value
Household with a parent %	30.8 ^2^	100	<0.0001
Household food security status ^3^ %			
Food secure and moderately food insecure	69.6	76.4	<0.0001
Severe food insecurity	30.4	23.6	
Grandparent with chronic disease ^4^ %	88.8	N/A	
Mother’s age (mean ± SE)	35.1 ± 1.06	38.9 ± 0.23	<0.0001
Mother’s Employment status %			
Employed	34.7	39.1	<0.0001
Unemployed	65.7	60.3	
Mother’s Educational attainment %			
Illiterate	22.9	23.6	<0.0981
Completed primary education	6.3	5.7	
Above primary education	70.8	70.7	
Father’s age ^5^ (mean ± SE)	39.9 ± 0.92	44.2 ± 0.25	<0.0001
Father’s Employment status %			
Employed	81.3	79.4	<0.08021
Unemployed	18.7	20.6	
Father’s Educational attainment %			
Illiterate	33.5	32.9	0.0200
Completed primary education	8.0	6.6	
Above primary education	58.6	60.6	
Household Demographic and Socio-economic status			
Households in poverty ^6^ %			
yes	71.1	67.3	0.0400
No	28.9	32.7	
Households receiving welfare %			
Yes	42.8	39.7	0.0120
No	57.2	60.3	
Household size (mean ± SE)	6.2 ± 0.16	5.5 ± 0.04	<0.0001
Number of children (mean ± SE)	2.2 ± 0.10	2.5 ± 0.03	0.0087
Number of adults (mean ± SE)	4.1 ± 0.12	3.2 ± 0.04	<0.0001
Household health expenditure per capita, in dollar (mean ± SE)	21.6 ± 3.00	13.6 ± 0.60	<0.0001
Household food expenditure per capita, in dollar (mean ± SE)	52.8 ± 2.28	58.8 ± 0.76	0.0004
Food-related assets ^7^ (mean ± SE)	2.3 ± 0.05	2.2 ± 0.01	0.0255

* Weighted percent. ^1^ Significant differences in proportions were tested using the chi-square test for categorical variables and the t-test for continuous variables, with the Bonferroni method of correction for multiple comparisons also being used. ^2^ This percentage (30.8%) indicates households with GP and with a parent in the household. ^3^ Household food security status was classified as: a score of 0–4 indicates food secure and moderately FI, and a score of 5–6 indicates being severely FI for 2010 survey (score 0–5 as food secure and moderately FI and 6–7 as severely FI for 2015 survey). ^4^ GP with chronic conditions: either grandparent who self-reported one or more chronic conditions. ^5^ Missing data on father in the household with live-in GP (n =29) and 104 households had an absent father. Missing data on father in the household without a live-in GP (n =53) and 114 households had an absent father. ^6^ Poverty status was based on household consumption expenditure equivalent to minimal food and non-food livelihood requirements per adult. The poverty line used was $6 per day [4,6]. ^7^ Food-related asset scale was calculated based on the ownership of refrigerator, freezer, oven, and microwave; each contributes one point to the scale.

**Table 3:** Sociodemographic and economic characteristics of children living in a household with and without a live-in grandparent (GP), (2010 and 2015 Socio-Economic Surveys of Palestinian Refugees in Lebanon). * ^1^.

Characteristics of Children	2010 and 2015 Surveys (n = 8034)		
	Children with Live-In GP 9.1%	Children without Live-In GP 90.9%	*p*-Value
Presence of Parent	69.5	100	<0.0001
Presence of GP only	30.5	N/A	
Household food security status ^2^ %			
Food secure and moderately food insecure	64.2	66.4	0.0002
Severe food insecurity	35.8	33.6	
Age (mean ±SE)	8.0 ± 0.2	9.5 ± 0.08	<0.0001
Age groups %			
0–4 years	28.2	21.1	<0.0001
5–11	45.5	38.6	
12–17	26.4	40.4	
Sex %			
Boys	49.4	50.3	0.8059
Girls	50.6	49.7	
Household poverty status %			
Yes	74.7	71.4	0.0390
No	25.3	28.6	
Having health insurance %			
Yes	53.0	54.8	0.0447
No	47.0	45.2	

* Weighted percent. ^1^ Significant differences in proportions were tested using the chi-square test for categorical variables and the *t*-test for continuous variables, with the Bonferroni method of correction being used for multiple comparisons. ^2^ Household food security status classified as: a score of 0–4 indicates being food secure and moderately FI, and a score of 5–6 is considered as severely FI for the 2010 survey (score 0–5 as food secure and moderately FI and 6–7 as severely FI for 2015 survey).

**Table 4:** Multivariate logistic regressions of factors associated with child’s health (0–17 years) (2010 and 2015 Socio-Economic Surveys of Palestinian Refugees in Lebanon). ^1^.

Characteristics	Acute Illness	Chronic Disease
	n = 7472	n = 7501
	OR (95% CI)	
Presence of Grandparent		
Yes vs. No	0.74 (0.62–0.92)	1.43 (0.46–2.45)
Presence of Parent		
Yes vs. No	0.73 (0.40–0.93)	0.82 (0.42–1.63)
Household food security status ^2^		
Severe food insecure vs. food secure and moderately food insecure	1.45 (1.17–1.80)	1.56 (1.19–2.07)
Child’s gender		
Female vs. Male	0.87 (0.82–0.93)	0.75 (0.65–0.87)
Child’s age groups		
0–4	1.35 (1.16–1.57)	0.61 (0.49–0.76)
5–11	1	1
12–17	1.02 (0.90–1.16)	0.98 (0.82–1.17)
Child has Health insurance		
Yes vs. No	1.13 (0.81–1.57)	0.87 (0.52–1.47)
Grandparent with chronic disease ^3^		
Yes vs. No	1.58 (0.42–2.98)	0.71 (0.21–2.38)
Household receiving welfare		
Yes vs. No	1.39 (1.17–1.65)	1.54 (1.22–1.94)
Health Expenditure Per Capita/(per $50)	1.42 (1.18–1.26)	1.45 (1.14–1.86)
Household size	0.89 (0.84–0.95)	0.91 (0.84–0.98)

^1^ All models were adjusted for the variables in the table and for survey year and household ID as fixed effect. ^2^ Household food security status was classified as: a score of 0–4 indicates food secure and moderately FI, and a score of 5–6 is considered as severely FI for the 2010 survey (0–5 as food secure and moderately FI and 6–7 as severely FI for 2015 survey). ^3^ GP with chronic disease: any grandparents who self-reported one or more chronic health condition.

**Table 5:** Multivariate logistic regressions of factors associated with child (5–17 years) wellbeing in Palestinian refugee camps in Lebanon (2010 and 2015 Socio-Economic Surveys of Palestinian Refugees in Lebanon). ^1^.

	School Attendance	Child Labor
	n = 5353	n = 4682
Presence of Grandparent in household		
Yes vs. No	2.22 (1.28–5.33)	1.61 (0.73–2.16)
Food security status 2		
Severe food insecurity vs. food security and moderate food insecurity	0.68 (0.35–0.71)	1.83 (1.30–2.78)
Presence of at least one parent in household		
Yes vs. No	1.75 (1.59–2.20)	0.51 (0.46–0.69)
Child’s gender		
Female vs. Male	1.44 (1.04–1.98)	0.72 (0.59–0.89)
Child’s age group for attending school		
6–12 years old vs. 12–17 years old	3.67 (2.41–4.64)	
Child age group		
5–14 years old vs. 15–17 years old		0.27 (0.18–0.41)
Grandparent with a chronic condition 3		
Yes vs. No	0.84 (0.19–1.61)	1.61(0.42–2.21)
Household receiving welfare		
Yes vs. No	1.37 (1.07–1.94)	0.62 (0.46–0.83)
Health Expenditure Per Capita/(per $50)	0.81 (0.66–0.99)	1.10 (0.78–1.54)
Household size	0.92 (0.82–1.04)	1.95 (1.86–2.04)

^1^ All models were adjusted for the variables in the table and for survey year and household ID as fixed effect. ^2^ Household food security status was classified as: a score of 0–4 indicated food secure and moderately FI, and a score of 5–6 was considered as severely FI for 2010 survey (0–5 as food secure and moderately FI and 6–7 as severely FI for 2015 survey). ^3^ GP with chronic conditions: any grandparent who self-reported one or more chronic condition.

## Data Availability

Restrictions apply to the availability of these data. Data are owned by the United Nations Relief and Works Agency for Palestinian Refugees in the Near East (UNRWA).
